# Does vitamin D have an effect on osseointegration of dental implants? A systematic review

**DOI:** 10.1186/s40729-022-00414-6

**Published:** 2022-04-11

**Authors:** Joscha G. Werny, Keyvan Sagheb, Leonardo Diaz, Peer W. Kämmerer, Bilal Al-Nawas, Eik Schiegnitz

**Affiliations:** 1 Department of Oral and Maxillofacial Surgery, University Medical Center of the Johannes-Gutenberg University, Augustusplatz 2, 55131 Mainz, Germany; 2 Postgraduate School, Faculty of Dentistry, Universidad de Chile, Santiago, Chile

## Abstract

**Purpose:**

The aim of this study was to systematically review the available evidence to evaluate the efficacy of vitamin D supplementation or vitamin D depletion on the osseointegration of implants in animals and humans.

**Methods:**

The focus questions addressed were “Do vitamin D deficient subjects treated with (dental) implants have an inferior osseointegration than subjects with adequate serum vitamin D level?” and “Do vitamin D supplemented subjects treated with (dental) implants have a superior osseointegration than subjects with adequate serum vitamin D level?” Humans and animals were considered as subjects in this study. Databases were searched from 1969 up to and including March 2021 using different combination of the following terms: “implant”, “bone to implant contact”, “vitamin D” and “osseointegration”. Letters to the editor, historic reviews, commentaries and articles published in languages other than English and German were excluded. The pattern of the present systematic review was customize to primarily summarize the pertinent data.

**Results:**

Thirteen experimental studies with animals as subject, two clinical studies and three case reports, with humans as subjects, were included. The amount of inserted titanium implants ranged between 24 and 1740. Results from three animal studies showed that vitamin D deficiency has a negative effect on new bone formation and/or bone to implant contact (BIC). Eight animal studies showed that vitamin D supplementation has a enhancing effect on BIC and/or new bone formation around implants. Furthermore, enhancing the impact of vitamin D supplementation on the osseointegration of implants in subjects with diabetes mellitus, osteoporosis and chronic kidney disease (CKD) were assessed. Studies and case reports involving human subjects showed that patients with a low serum vitamin D level have a higher tendency to exhibit an early dental implant failure. When supplemented with vitamin D the osseointegration was successful in the case reports and a beneficial impact on the changes in the bone level during the osseointegration were determined.

**Conclusions:**

Vitamin D deficiency seems to have a negative effect on the osseointegration of implants in animals. The supplementation of vitamin D appears to improve the osseointegration in animals with systemic diseases, such as vitamin D deficiency, diabetes mellitus, osteoporosis, and CKD. Slight evidence supports the hypothesis that humans similarly benefit from vitamin D supplementation in terms of osseointegration. Further investigation is required to maintain these assumptions.

## Introduction

Successful osseointegration is one of the key criteria for a prosperous dental implant therapy which is achieved by a functional ankylosis. The foreign material and vital bone grow together as a functional unit which is characterized as the initial newly created bone to implant contact (BIC) [1, 2]. New bone formation can be both enhanced [3] or decelerated [4, 5] due to vitamin D, depending on its level. Vitamin D deficiency is associated with a variety of diseases, such as parodontitis [6, 7], early tooth loss [8], a catabolic metabolism, osteoporotic fractures [5] and compromised fracture healing [9, 10]. Vitamin D is a steroid hormone which can be synthesized in the skin when sun irradiation is sufficient (290–315 nm) and successfully converted in the liver and kidneys [11]. Sufficient sun irradiation is only available in months from April until September northern 40° N latitude, which is northern from Madrid [12]. Therefore, most humans living in Europe, northern Asia and North America are not adequately supplied with vitamin D throughout the year from sunlight alone. Vitamin D is additionally contained in nourishment, such as oily fish, liver (beef), eggs, milk, cheese soy and mushrooms [13, 14]. It plays an important role in the mineral homeostasis by stimulating intestinal absorption of calcium and phosphate [15]. It also regulates the bone metabolism and bone mineralization by activating osteoclasts and osteoblasts [5, 16, 17].

**Fig. 1:**
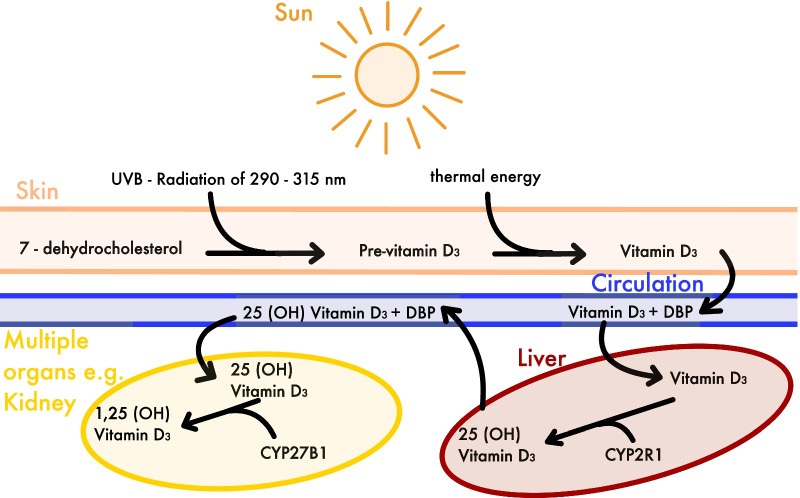
Endogenous synthesis of 1,25 (OH) Vitamin D. DBP: Vitamin D-binding Protein, CYP2R1: Vitamin D 25-hydroxylase, CYP27B1: 1 alpha-hydroxylase

Vitamin D is available in different sources and, therefore, has different versions. Ergocalciferol (Vitamin D_2_) is the versions contained in plants and Cholecalciferol (Vitamin D_3_) in animals and humans [18, 19] (Fig. 1). There are also active and inactive versions of vitamin D depending on the way of hydroxylation. For laboratory measurements 25 (OH) vitamin D status is determined, since it is a metabolite with a high concentration and a relatively long half-life. The active version, however, is 1,25 (OH) vitamin D. Since the laboratory measurement detects only the level of 25 (OH) vitamin D and solely in the bloodstream it is not a perfectly accurate method to measure all disposable vitamin D inside the body. However, it is a good measurement of the available vitamin D in the bloodstream and the established marker of vitamin D status [19, 20]. Furthermore, multiple laboratory measurements are available to determine the concentration of other vitamin D metabolites, but none of them were part of studies included in this systematic review.

Schulze-Späte et al. [21] assessed the biological effect of systemic vitamin D supplementation on bone cells. This randomized, double-blind, placebo-controlled clinical investigation showed that patients supplemented with vitamin D demonstrate a higher osteoclast activity compared with control. Both positive [22–27] and negative [28] effects of vitamin D supplementation on BIC have been mentioned in the previous literature. Liu et al. [24] and Zhou et al. [25] for instance showed a significant enhanced bone volume and osseointegration compared with control. However, Naito et al. [29] and Akhavan et al. [30] did not show a significant effect of vitamin D supplementation in terms of increased BIC. Concerning vitamin D deficiency, all studies [23, 31, 32] showed a significant decreasing BIC or new bone formation compared to control or vitamin D supplemented rats. There seems to be a disagreement over the efficacy of vitamin D supplementation concerning the augmenting BIC. Hence the aim of this study was to systematically review the available evidence to evaluate the efficacy of vitamin D supplementation or vitamin D depletion on the osseointegration of implants.

## Materials and methods

### Focused questions

**Fig. 2:**
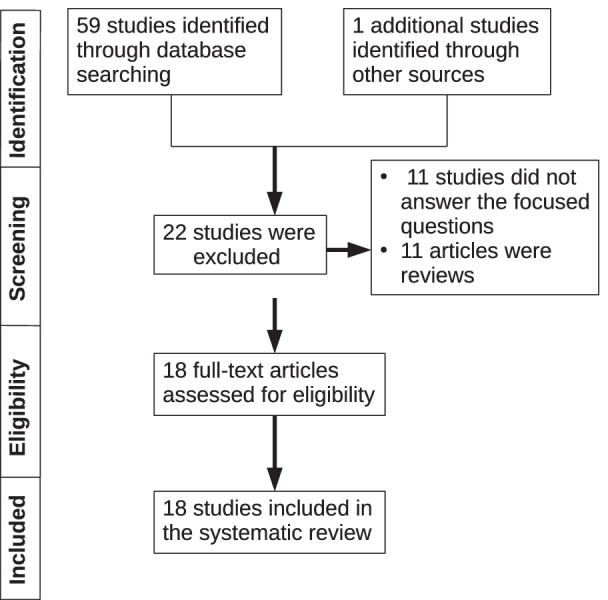
Flow chart showing the search strategy that was adopted to identify studies that fulfilled the eligibility criteria. This flow chart was constructed in accordance with the Preferred Reporting Items for Systematic Review and Meta-Analysis guidelines

Based on the preferred reporting items for systematic reviews and meta-analysis (PRISMA) guidelines, two particular questions were created according to the participants, intervention, control, outcomes (PICO) principle (Fig. 2). The constructed Questions were:

1. Do vitamin D deficient subjects treated with (dental) implants have an inferior osseointegration than subjects with adequate serum vitamin D level and.
2. do vitamin D supplemented subjects treated with (dental) implants have a superior osseointegration than subjects with adequate serum vitamin D level?

(1)

(P) Participants: it was crucial for subjects (animals or humans) to have undergone implant treatment.

(I) Types of interventions: the intervention of interest was the effect of vitamin D deficiency on osseointegration.

(C) Control intervention: osseointegration with adequate vitamin D serum level.

(O) Outcome measures: bone to implant contact (BIC), new bone formation (NBF) and/or Implant resistance, bone volume/tissue volume (BV/TV) around the implants with and without vitamin D deficiency.

(2)

(P) Participants: it was crucial for subjects (animals or humans) to have undergone implant treatment.

(I) Types of intervention: the intervention of interest was the effect of vitamin D supplementation on osseointegration.

(C) Control intervention: osseointegration with adequate vitamin D serum level.

(O) Outcome measures: bone to implant contact (BIC), new bone formation (NBF) and/or Implant resistance, bone volume/tissue volume (BV/TV) around the implants with and without vitamin D supplementation.

### Eligibility criteria

The eligibility criteria were as follows: (a) original studies or case reports (clinical and experimental), (b) inclusion of a control group (osseointegration around implants without vitamin D supplementation/deficiency) (c) intervention: effect of vitamin D supplementation/deficiency on osseointegration (d) conservative non-intervention studies and (e) studies published in English and German language. Letters to the editor, historic reviews and commentaries were excluded.

### Literature search protocol

PubMed/Medline (National library of Medicine, Washington, DC, USA), Cochane library and Google-Scholar databases was searched from 1969 to March 2021 using different combination of the following terms: “implant”, “bone to implant contact”, “vitamin D” and “osseointegration”. Titles and abstracts of studies identified using the above-described protocol were screened by the author (JW). Full texts of studies judged by title and abstract to be relevant were read and independently evaluated for the stated eligibility criteria. Reference lists of potentially relevant original and review articles were hand searched to identify any studies that could have remained unidentified in the previous step. The pattern of the present systematic review was customized to mainly summarize the relevant data. The initial search yielded 60 studies. Forty two studies which did not fulfill the eligibility criteria were excluded. In total, 18 experimental [22–35] (Table 1) and non-intervention clinical studies [36] and case reports [37–39] were included and processed for data extraction (Table 2).

**Table 1:** Characteristics (authors, study design and subject, geometry and location of implant placement, study groups, follow-up period and outcomes) of studies with animal subjects included in the present systematic review

Authors	Study design	Study subjects	Number of implans, *n*	Implant dimension Ø × *L* in mm	Implant location	Study groups, *n* = subjects	Follow-up	Outcome
Kelly et al. [31]	Animal study	14 rats	28 Ti Implants	1 × 2	Femur	Group-1 (*n* = 7): vitamin d deficient Group-2 (*n* = 7): control	2 weeks	Group-1 has significant decreased push in test and BIC compared to control
Vandersteenhoven et al. [32]	Animal study	56 rats	168 Implants	NA	Abdominal region	Group-1 (*n* = 27): vitamin D_3_ supp Group-2 (*n* = 24): control Group-3 (*n* = 5): VD3 supp	1, 2, 3 and 10 weeks	Group-2 showed less remodeling, NBF and bone resorption than Group-1
Pimentel et al. [33]	Animal study	36 rats	72 Ti implants	2.5 × 4	Tibia	Group-1 (*n* = 18): control Group-2 (*n* = 18): Ca, vit D, Mg and Zn supp	30 days	No significant differences in BIC or NBF between the groups
Wu et al. [22]	Animal study	30 rats	N/A Ti	1 × 10	Femur	Group-1 (*n* = 6): control; Group-2 (*n* = 6): diabetic; Group-3 (*n* = 6): insulin-treated diabetic; Group-4 (*n* = 6): VD3-treated diabetic; Group-5 (*n* = 6): VD3 + insulin-treated	12 weeks	Group-5 had significant higher BIC, BV/TV and BA compared to Group-2 and similar to Group-1
Akhavan et al. [30]	Animal study	48 rats	N/A Ti	1.2 × 7	Tibia	Group-1 (*n* = 17): VD3 supp Group-2 (*n* = 10): control	3 and 6 weeks	No significant difference in BIC or NBF between the groups
Liu et al. [24]	Animal study	30 mice	60 Ti implants	1 × 4	Femur	Group-1 (*n* = 10): control Group-2 (*n* = 10): nephrectomy Group-3 (*n* = 10): nephrectomy and VD3 supp	2 weeks	Group-3 had significantly higher BIC and BV/TV around implant than Group-1 and -2. Group 3 had significant higher resistance of implants compared to Group-2
Dvorak et al. [23]	Animal study	50 rats	100 Ti Implants	1 × 3	Tibia	Group-1 (*n* = 16): VD3 depletion Group-2 (*n* = 17): VD3 repletion 6 Group-3 (*n* = 17): controls	4 weeks	Group-1 had significant less cortical BIC compared to Group-3 Group-2 had significant decreased BV/TV compared to Group-1 and -3
Zhou et al. [25]	Animal study	20 rats	40 Ti Implants	1.8 × 3,5	TIbia	Group-1 (*n* = 10): VD3 supp Group-2 (*n* = 10): control	8 weeks	Group-1 had significant increased BV/TV, BIC, BA and implant resistance compared to Group-2
Nakamura et al. [34]	Animal study	64 rats	128 Ti Implants	1.4 × 23	Femur	Group-1 (*n* = 8): control Group-2 (*n* = 8): ovariectomized Group-3 (*n* = 16):VD3 supp Group-4 (*n* = 16): alendronate Group-5 (*n* = 16): alendronate and VD3 supp	4 weeks	Group-5 had significant higher BA in compared to Group-2 Group-5 showed higher resistance compared to control after 12 weeks of supp
Satué et al. [28]	Animal study	6 rabbits	24 Ti implants	6.25 × 2	Tibia	Grooup-1 (*n* = 8): control Group-2 (*n* = 8): low dose 7-DHC and vit. E coated implants Group-3 (*n* = 8): high dose 7-DHC and vit.E coated implants	8 weeks	No significant differences in BIC or NBF between the groups
Naito et al. [29]	Animal study	12 rabbits	28 Ti implants	3.75 × 7	Tibia	Group-1 (*n* = 7): control Group-2 (*n* = 7): coated with VD3 c = 10^–8^ M (mol/L) Group-3 (*n* = 7): c = 10^–7^ M (mol/L) Group-4 (*n* = 7): c = 10^–6^ M (mol/L)	6 weeks	No significant differences in BIC or NBF between the groups
Salomó-Coll et al. [26]	Animal study	6 dogs	24 Ti implants	3,75 × 10	Mandibula	Group-1 (*n* = 12): control Group-2 (*n* = 12): VD2 coated implants	12 weeks	Group-2 had significant higher total BIC and NBF in compared to control
Cho et al. [27]	Animal study	12 rabbits	48 Ti implants	3.75 × 7	Tibia	Group-1 (*n* = 6): coated with PLGA/VD3 solution Group-2 (*n* = 6): control	4 and 12 weeks	Group 1 had significant greater BIC compared to Group-2

VD3:1,25(OH)_2_ D3; 25 (OH): 25 (OH) Hydroxyvitamin D; VD2: Ergocalciferol; 7-DHC: 7-dehydrocholesterol; vit. E: vitamin E; BIC: bone to implant contact; NBF: New bone formation; BV/TV: Bone volume/Tissue volume; LHD: Lactate dehydrogenase; Supp.: Supplementation, N/A: not available BA: bone area density

**Table 2:** Characteristics (authors, study design and subject, geometry and location of implant placement, study groups, follow-up period and outcomes) of studies and case reports with human subjects included in the present systematic review

Authors	Study design	Study subjects	Number of implans, *n*	Implant Dimension Ø × *L* in mm	Implant Location	Study groups, *n* = subjects	Follow-up	Outcome
Flanagan et al. [37]	Case report	1 Man	18 Implants	N/A	Mandible & maxilla	Patient with ESRD VD3 supplementation, phosphate binders and calcium cinacalcet calcimimeti, 3 × dialysis/week	7 months	Successful immediate implantation after extraction
Bryce et al. [38]	Case report	1 Man	1 Ti Implant	4.3 × 10	Mandible	Patient with serve vitamin D deficiency (25 OH vitamin D < 10 nmol/L)	5 months	Failed immediate implantation after extraction due to missing osseointegration
Fretwurst et al. [39]	Case report	2 Men	9 TiZr Implants	4.1 × 12–4.3 × 7	Mandible	Healthy patients with vitamin D deficiency	ND	Implant placement failed once/twice, after VD3 supp. implant placement was successful
Guido Mangano et al. [36]	Human retrospective study	885 Humans	1740 implants	N/A	Mandible and maxilla	Group-1 (*n* = 48): < 10 ng/mL 25 (OH) Group-2 (*n* = 448): 10–30 ng/mL 25 (OH) Group-3 (*n* = 410): > 30 ng/mL 25 (OH)	4 months	Tendency to more EDIF the lower the 25 (OH) vitamin d level Group-1: 11,1% EDIF; Group-2 4,4% EDIF; Group-3: 2,9%
Kwiatek et al. [35]	Human prospective study	122 Humans	122 Ti implants	3,3–4,2 × 8–11,5	Mandible	Group-1: (*n* = 43): < 30 ng/ml 25 (OH) Group-2: (*n* = 48): D < 30 ng/ml 25 (OH) + supp of VD3 Group-3 (*n* = 31): > 30 ng/ml 25 (OH)	6 and 12 weeks	Group-2 had significant higher bone level changes around dental implants compared to Group-1, after 12 weeks

VD3:1,25(OH)_2_ D3; 25 (OH): 25 (OH) Hydroxyvitamin D; Supp.:Supplementation; EDIF: Early dental implant failure; ESRD: end-stage renal disease, N/A: not available

## Results

### Results of studies carried out with animals

#### General characteristics

The majority [22–34] of the studies were performed with animals. Few studies [35, 36] and case reports [37–39] were performed with humans, respectively. One study [24] was performed with mice. Eight studies [22, 23, 25, 30–34] were performed with rats, of which six studies involved only one sex. Each sex was included three times separately, male [22, 32, 33] and female [23, 25, 34] rats. In two [30, 31] studies the sex of the used rats was not reported. Three studies [27–29] included rabbits of which one [28] was performed with females only. One study [26] was performed with dogs. In almost all animal studies [22–31, 33, 34] Ti implants were used, one study [32] was performed with Demineralized Allogeneic Bone Matrix (DABM) as implant material. The number of inserted implants ranged between 24 and 168. The number of placed implants was not reported in two studies [22, 29]. Dimensions (diameter × length in millimeters) ranged between 1 × 2, 6.25 × 2 and 1.4 × 23. Implants were placed in tibia, femur and jaw in seven [23, 25, 27–30, 33], four [22, 24, 31, 34] and one study [26], respectively. In the study by Vandersteenhoven et al. [32] the region of the implantation was subcutaneously in abdominal sites. The follow-up period of the studies ranged between 1 and 8 weeks. In four studies [26–29] vitamin D coated and non-coated implants were evaluated on their impact on osseointegration. Moreover, the effect of vitamin D supplementation on osseointegration was assessed in ovariectomized rats [23, 25, 34], diabetic rats [22, 30], nephrectomized mice [24] and vitamin D deficient rats [23, 31, 32].

#### Assessment of osseointegration

In eleven studies [22–27, 29–32, 34] histological analysis was used to assess the osseointegration. In six studies [22, 25, 28, 31, 33, 34] the implant resistance was assessed due to biomechanical tests, the new bone formation and strength of the newly formed bone around the implant were measured. In three studies [22, 25, 33] the new bone formation around implants was assessed using a three-dimensional (3D) microscopic computerized tomography (micro-CT), two studies [24, 26] used conventional two-dimensional (2D) radiography to verify implant osseointegration and to assess changes in post-surgical crestal bone levels, respectively. In seven studies [22–27, 29, 32] histomorphometry was used to assess the osseointegration around the implants and scanning electron microscope (SEM) was used in two studies [31, 34]. Wound fluid analysis was performed in the study by Satué et al. [28] to assess the cellular activity while osseointegration. Serum vitamin D level have been measured in one study [22].

#### Main outcome

**Fig. 3:**
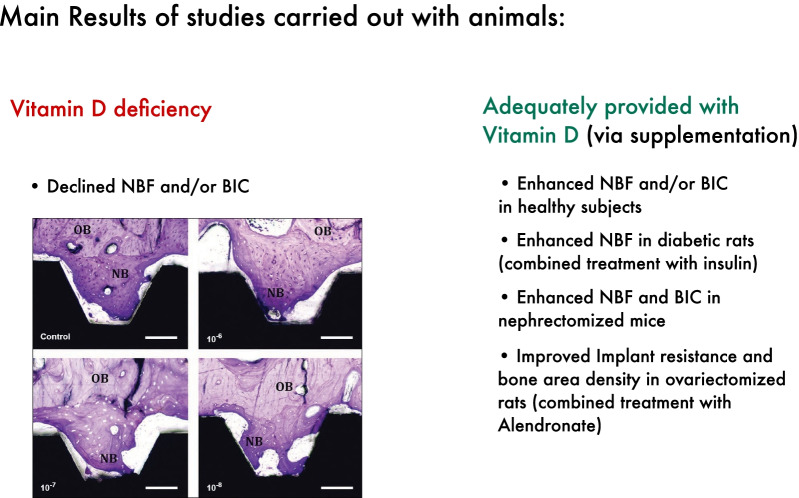
Overview concerning the main results of studies carried out with animals. NBF: New bone formation, BIC: Bone to implant contact. Including histological pictures published by Naito et al. 2014 (https://www.ncbi.nlm.nih.gov/pmc/articles/PMC4219862/), no changes have been made to the Figure. Copyright^©^ Naito Y, Jimbo R, Bryington MS, Vandeweghe S, Chrcanovic BR, Tovar N, Ichikawa T, Coelho PG, Wennerberg A. (http://creativecommons.org/licenses/by-nc-nd/3.0/)

The results of three animal studies [23, 31, 32] showed that vitamin D deficiency declined new bone formation and/or BIC around implants. Eight studies [22–27, 32, 34] involving animals provided adequately with vitamin D showed that vitamin D supplementation enhanced new bone formation and/or BIC around implants. Four studies reported no statistically significant differences in BIC and/or new bone formation around vitamin D coated implants [28, 29] or systemic vitamin D supplementation [30, 33]. Diabetic rats had an improved osseointegration when treated with insulin [22]. Additional vitamin D supplementation enhanced the new bone formation around implants in diabetic rats [22], while vitamin D supplementation solely had no significant statistical effect [30]. A different study [24] reported significant enhanced new bone formation and BIC in nephrectomized mice when supplemented with vitamin D. Significant improvement in implant resistance and bone area density was achieved in ovariectomized rats after treatment with a combination of vitamin D and Alendronate [34] (Fig. 3).

### Results of clinical studies

#### General characteristics

Humans were subjects in two studies [35, 36] and three case reports [37–39]. The case reports were developed with male patients only. In all studies and case reports [35–39] humans had undergone dental implant treatment with Ti [35–38] or TiZr [39] implants. The number of inserted implants ranged between 4 and 1740. Dimensions (diameter × length in millimeters) ranged between 3.3 mm × 8 mm, 4.1 mm × 12 mm and 4.3 mm × 7 mm. The follow-up period of the studies ranged between 12 weeks and 7 months. One study [35] evaluated the effect of vitamin D supplementation on changes in the bone level at implant site during the process of osseointegration. A different study [36] aimed to discover the ratio of early dental implant failures (EDIF = i.e., failure that occurs within 4 months after placement, before the connection of the prosthetic abutment) in correlation with the serum 25 (OH) vitamin D level. Moreover, the effect of vitamin D supplementation on osseointegration was assessed in human patients with end-stage renal disease (ESRD) [37] or vitamin D deficiency [38, 39] by the case reports.

#### Assessment of osseointegration

Kwiatek et al. [35] used conventional two-dimensional (2D) radiography to verify implant osseointegration and to assess changes in post-surgical crestal bone levels, respectively. In addition, they used ISQ (Implant stability quotient) measurement to assess the implant stability due to osseointegration. Serum vitamin D level have been measured in four studies [35, 36, 38, 39].

#### Main outcome

Two case reports [38, 39] with human subjects described those three patients have been losing their dental implant while having a vitamin D deficiency. Mangano et al. [36] discovered that patients with a low serum vitamin D level have a higher tendency to exhibit an EDIF.

**Fig. 4:**
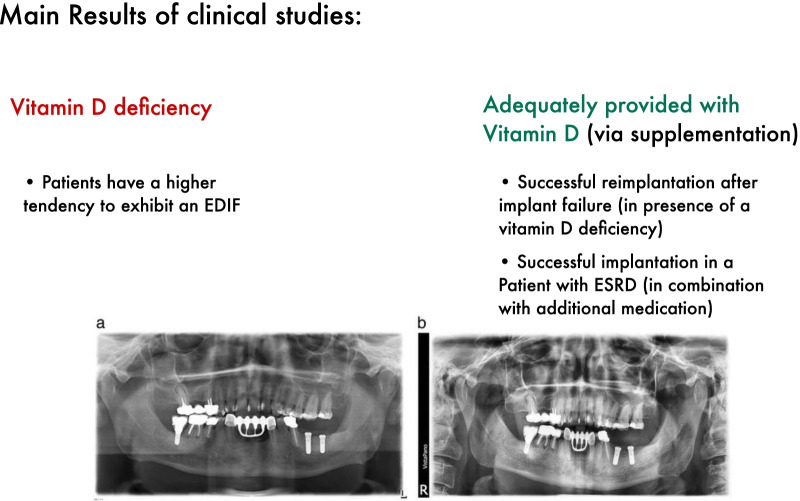
Overview concerning the main results of clinical studies. ESRD: End-stage renal failure, EDIF: Early dental implant failure. Including orthopantomogram pictures published by Fretwurst et al. 2016 (https://link.springer.com/content/pdf/10.1186/s40729-016-0056-0.pdf), no changes have been made to the figure. Copyright^©^ Tobias Fretwurst, Sebastian Grunert, Johan P. Woelber, Katja Nelson and Wiebke Semper-Hogg (http://creativecommons.org/licenses/by/4.0/).

Kwiatek et al. [35] showed in their study that vitamin D supplementation has an increasing influence on the change of bone level around dental implants. Fretwurst et al. [39] reported two cases in which vitamin D deficient patients have been successfully treated with dental implants after one or two consecutive dental implant failures, due to vitamin D supplementation. Flanagan et al. [37] reports one case in which a patient with ESRD was successfully treated with dental implants due to vitamin D supplementation and additional medication (Fig. 4).

## Discussion

From the literature reviewed, 18 studies [22–39] fulfilled our eligibility criteria; however, results from all animal studies [23, 31, 32] which compared vitamin D deficient to non-deficient subjects showed that BIC or new bone formation have been declined. According to the Institute of medicine (IOM) approximately 20–100% of the adult women and men living in northern Europe and North America appear to have vitamin D deficiency [5, 40–44]. Therefore, this finding seems to have a high clinical relevance on dental treatment in these regions.

The result from 75% [22–25, 32, 34] of the studies showed that systemically vitamin D supplementation enhanced new bone formation around dental implants. However, Pimentel et al. [33] and Akhavan et al. [30] showed no significant difference of new bone formation or bone to implant contact in subjects with systematically vitamin D supplementation. In fact, the study of Pimentel et al. [33] has been the only study with healthy systematically vitamin D supplemented animals. The supplementation of healthy subjects with vitamin D has only a weak improving effect. Vitamin D sufficient or insufficient patients benefit less of a vitamin D supplementation than deficient patients [45]. In healthy patients a high-dose vitamin D supplementation can cause a lower bone mineral density [4].

Two studies [22, 30] have assessed the effect of vitamin D supplementation on osseointegration of diabetic rats. Both couldn’t show a statistically significant difference between vitamin D supplemented and not supplemented rats regarding BIC and new bone formation. The combined treatment of insulin and vitamin D supplementation [22] led to a significant higher BIC and BA compared to not treated diabetic rats and similar results as rats in the healthy control group. However, the supplementation of vitamin D in pre-diabetic, vitamin D deficient patients could reduce the risk of developing type 2 diabetes by 62% [45].

The period of vitamin D supplementation in the animal studies ranged between 7 day post-surgery and 8 week pre-surgery and 4 week post-surgery. Most studies [22, 25, 30, 33, 34] started post-surgery with the vitamin D supplementation. Only three studies [20, 21, 31] started pre-surgery. Dvorak et al. [23] and Nakamura et al. [34] assessed the effect of different supplementation periods on the osseointegration. Dvorak et al. [23] discovered that the deficient group had significantly less cortical BIC compared to the group which was supplemented 8 week pre-surgery and 14 day post-surgery. A different group which was supplemented 14 day pre-surgery and post-surgery had no significant change in BIC; however, the BV/TV was significantly decreased compared to the two other groups. Nakamura et al. [34] could not show a statistically significant difference between pre-surgery or post-surgery supplemented, ovariectomized rats. Neither in animals nor in humans there is a common recommendation on how and when to supplement or measure the 25 (OH) vitamin D level [46, 47].

Analog to animals, humans also showed a negative effect of low vitamin D levels on the osseointegration of dental implants. Mangano et al. [36] discovered that the number of EDIF’s has a tendency to increase when the patients show lower vitamin D levels. This finding leads to the hypothesis that humans similar to animals may also have an inferior osseointegration of implants due to vitamin D deficiency compared with subjects with an adequate vitamin D level. Furthermore, Kwiatek et al. [35] showed that systemically vitamin D supplementation enhanced new bone formation around dental implants. This result is similar to most of the studies carried out with animals and is slight evidence for a commonality with animals in this feature. All case reports [37–39] support the assumption that human beings have comparable symptoms when vitamin D deficient, supplementation of vitamin D seems to reduce these symptoms and help patients with a disturbed mineral homeostasis.

Only 50% of the animal studies [26, 27] showed that local vitamin D supplementation enhanced the bone to implant contact. The other two studies [28, 29] did not show significant difference in bone to implant contact, though on a cellular level there was a certain difference. Satué et al. [28] reported a significantly higher level of LHD around implants coated with a high dose of UV-irradiated vitamin D precursor and vitamin E compared to control. However, the low dosed coated implants had a significantly higher level of ALP (Alkaline phosphatase) compared with the high dosed coated implants. The osteocalcin level was significantly higher in the group of low doses coated implants. These results lead to the hypothesis that different concentrations of implant coatings have oppositional effects on the surrounding bone tissue.

It is relevant to mention that the majority of the studies [22–34] which assessed the effect of vitamin D deficiency or supplementation on osteogenesis around implants were performed in animals and the used methods remarkably varied among the studies included. The subjects in which the osseointegration around implants were assessed were mice, rats, rabbits and dogs. Some studies relied on histological analysis [22–27, 29, 30, 32] to evaluate new bone formation, others based on micro-CT analysis [22, 25, 33], while different depend on SEM analysis [31, 34]. Micro-CTs have higher resolution than computed tomography used for patients and are able to measure the bone mineral density. Cancellous bone can be evaluated by means of the trabeculae characteristics and their connectivity [48]. This enables a far more distinct assessment of the bone and bone–implant interface. Histological analysis continues to be the gold standard for assessing bone formation on a cellular basis; however, they both provided comparable results for BIC, BA and bone–implant volume [49, 50].

Due to the very short follow-up period of the animal studies (up to 12 weeks) only little is known about the long-term effects of vitamin D supplementation on the survival rate of implants. Further studies are necessary to evaluate these long-term effects on the implant survival and may provide stronger evidence for the supporting effect of vitamin D on osseointegration. Animals with systemic diseases such as diabetes, osteoporosis and CKD did have a great benefit of BIC and/or new bone formation after a combined treatment with their standard medication plus vitamin D supplementation. Longitudinal, randomized controlled trials with human beings are needed to test whether this positive effect of vitamin D supplementation also applies on human beings and, therefore, improve the implant treatment of patients with these diseases.

Since there was just a small number of studies [35, 36] their results imply only weak evidence of the improvement on osseointegration due to vitamin D supplementation in humans. The case reports [37–39] investigating the effect of vitamin D on osseointegration in humans have a high risk of bias. Especially the effect of vitamin D supplementation on osseointegration in patients with diseases such as diabetes, osteoporosis, vitamin D deficiency and kidney diseases remains widely unknown.

## Conclusions

Within the limitation of the present systematic review, it is concluded that vitamin D deficiency seems to have a negative effect on the osseointegration of implants in animals. The supplementation of vitamin D appears to enhance the osseointegration in animals with systemic diseases, such as vitamin D deficiency, diabetes mellitus, osteoporosis and CKD. The effect of vitamin D coated implants on the osseointegration remains controversial. Only slight evidence supports the hypothesis that humans similarly benefit from vitamin D supplementation in terms of osseointegration. Further investigations on the effect of vitamin D on osseointegration of dental implants in humans are required to maintain these assumptions.

## Data Availability

The included studies are available within PubMed/Medline (National library of Medicine, Washington, DC, USA), Cochane library and Google-Scholar databases.

## Abbreviations

ALP: Alkaline phosphatase
BA: Bone area density
BIC: Bone to implant contact
BV/TV: Bone volume/tissue volume
CKD: Chronic kidney disease
DABM: Demineralized allogeneic bone matrix
EDIF: Early dental implant failures
ESRD: End-stage renal disease
IOM: Institute of medicine
ISQ: Implant stability quotient
J.W.: Joscha G. Werny
LHD: Lactate dehydrogenase
micro-CT: Microscopic, computerized tomography
NBF: New bone formation
PICO: Participants, intervention, control, outcomes
PRISMA: Preferred reporting items for systematic reviews and meta-analysis
SEM: Scanning electron microscope
Ti: Titanium
Zr: Zirconia

## References

[1] Brånemark PI, Adell R, Breine U, Hansson BO, Lindström J, Ohlsson A (1969). Intra-osseous anchorage of dental prostheses. I. Experimental studies. Scand J Plast Reconstr Surg.

[2] Schroeder A, van der Zypen E, Stich H, Sutter F (1981). The reactions of bone, connective tissue, and epithelium to endosteal implants with titanium-sprayed surfaces. J Maxillofac Surg Februar.

[3] Hong H-H, Yen T-H, Hong A, Chou T-A (2015). Association of vitamin D3 with alveolar bone regeneration in dogs. J Cell Mol Med Juni.

[4] Burt LA, Billington EO, Rose MS, Raymond DA, Hanley DA, Boyd SK (2019). Effect of high-dose vitamin D supplementation on volumetric bone density and bone strength: a randomized clinical trial. JAMA.

[5] Holick MF (2007). Vitamin D deficiency. N Eng J Med.

[6] Laky M, Bertl K, Haririan H, Andrukhov O, Seemann R, Volf I (2017). Serum levels of 25-hydroxyvitamin D are associated with periodontal disease. Clin Oral Invest.

[7] Antonoglou GN, Knuuttila M, Niemelä O, Raunio T, Karttunen R, Vainio O (2015). Low serum level of 1,25(OH)2D is associated with chronic periodontitis. J Periodontal Res.

[8] Zhan Y, Samietz S, Holtfreter B, Hannemann A, Meisel P, Nauck M (2014). Prospective study of serum 25-hydroxy vitamin D and tooth loss. J Dental Res..

[9] Brinker M, O’Connor D, Monla Y, Earthman T (2007). Metabolic and endocrine abnormalities in patients with nonunions. J Orthop Trauma.

[10] Alkalay D, Shany S, Dekel S (1989). Serum and bone vitamin D metabolites in elective patients and patients after fracture. J Bone Jt Surg Br.

[11] Lehmann B (2005). The vitamin D3 pathway in human skin and its role for regulation of biological processes. Photochem Photobiol.

[12] O’Connor A, Benelam B (2011). An update on UK Vitamin D intakes and status, and issues for food fortification and supplementation. Nutr Bull.

[13] Holick MF, Chen TC (2008). Vitamin D deficiency: a worldwide problem with health consequences. Am J Clin Nutr.

[14] U.S. Department of Agriculture, Agricultural Research Service. FoodData Central [Internet]. [zitiert 9. März 2021]. Verfügbar unter: https://fdc.nal.usda.gov/index.html.

[15] Halfon M, Phan O, Teta D. Vitamin D: a review on its effects on muscle strength, the risk of fall, and frailty. Biomed Res Int. 2015. Verfügbar unter: https://www.ncbi.nlm.nih.gov/pmc/articles/PMC4427016/. 10.1155/2015/953241 PMC4427016 26000306

[16] Kogawa M, Findlay DM, Anderson PH, Ormsby R, Vincent C, Morris HA (2010). Osteoclastic metabolism of 25(OH)-vitamin D3: a potential mechanism for optimization of bone resorption. Endocrinology.

[17] Atkins GJ, Kostakis P, Pan B, Farrugia A, Gronthos S, Evdokiou A (2003). RANKL expression is related to the differentiation state of human osteoblasts. J Bone Mineral Res..

[18] Ross AC, Manson JE, Abrams SA, Aloia JF, Brannon PM, Clinton SK (2011). The 2011 report on dietary reference intakes for calcium and vitamin D from the Institute of Medicine: what clinicians need to know. J Clin Endocrinol Metab.

[19] Nutrition and bone health: with particular reference to calcium and vitamin D. Report of the Subgroup on Bone Health, Working Group on the Nutritional Status of the Population of the Committee on Medical Aspects of the Food Nutrition Policy. Rep Health Soc Subj (Lond). 1998;49:iii–xvii, 1–24. PMID: 9932291. 9932291

[20] Makris K, Sempos C, Cavalier E (2020). The measurement of vitamin D metabolites: part I—metabolism of vitamin D and the measurement of 25-hydroxyvitamin D. Hormones.

[21] Schulze-Späte U, Dietrich T, Wu C, Wang K, Hasturk H, Dibart S (2016). Systemic vitamin D supplementation and local bone formation after maxillary sinus augmentation—a randomized, double-blind, placebo-controlled clinical investigation. Clin Oral Implants Res.

[22] Wu Y, Yu T, Yang X, Li F, Ma L, Yang Y (2013). Vitamin D3 and insulin combined treatment promotes titanium implant osseointegration in diabetes mellitus rats. Bone.

[23] Dvorak G, Fügl A, Watzek G, Tangl S, Pokorny P, Gruber R (2012). Impact of dietary vitamin D on osseointegration in the ovariectomized rat. Clin Oral Implant Res.

[24] Liu W, Zhang S, Zhao D, Zou H, Sun N, Liang X. Vitamin D supplementation enhances the fixation of titanium implants in chronic kidney disease mice. PLoS One. 9(4). Verfügbar unter: https://www.ncbi.nlm.nih.gov/pmc/articles/PMC3994107/. 10.1371/journal.pone.0095689 PMC3994107 24752599

[25] Zhou C, Li Y, Wang X, Shui X, Hu J (2012). 1,25Dihydroxy vitamin D3 improves titanium implant osseointegration in osteoporotic rats. Oral Surg Oral Med Oral Pathol Oral Radiol.

[26] Salomó-Coll O, Val de JEM-S, Ramírez-Fernandez MP, Hernández-Alfaro F, Gargallo-Albiol J, Calvo-Guirado JL (2016). Topical applications of vitamin D on implant surface for bone-to-implant contact enhance: a pilot study in dogs part II. Clin Oral Implants Res.

[27] Cho YJ, Heo SJ, Koak JY, Kim SK, Lee SJ, Lee JH (2011). Promotion of osseointegration of anodized titanium implants with a 1α,25-dihydroxyvitamin D3 submicron particle coating. Int J Oral Maxillofac Implants.

[28] Satué M, Monjo M, Ronold HJ, Lyngstadaas SP, Ramis JM (2017). Titanium implants coated with UV-irradiated vitamin D precursor and vitamin E: in vivo performance and coating stability. Clin Oral Implant Res.

[29] Naito Y, Jimbo R, Bryington MS, Vandeweghe S, Chrcanovic BR, Tovar N. The influence of 1α.25-Dihydroxyvitamin D3 coating on implant osseointegration in the rabbit Tibia. J Oral Maxillofac Res. 2014;5(3). Verfügbar unter: https://www.ncbi.nlm.nih.gov/pmc/articles/PMC4219862/. 10.5037/jomr.2014.5303 PMC4219862 25386230

[30] Akhavan A, Noroozi Z, Shafiei AA, Haghighat A, Jahanshahi GR, Mousavi SB (2012). The effect of vitamin D supplementation on bone formation around titanium implants in diabetic rats. Dent Res J (Isfahan).

[31] Kelly J, Lin A, Wang CJ, Park S, Nishimura I (2009). Vitamin D and bone physiology: demonstration of vitamin D deficiency in an implant osseointegration rat model. J Prosthodont.

[32] Vandersteenhoven JJ, DeLustro FA, Bell NH, Turner RT (1988). Osteoinduction by implants of demineralized allogeneic bone matrix is diminished in vitamin D-deficient rats. Calcif Tissue Int.

[33] Pimentel SP, Casarin RC, Ribeiro FV, Cirano FR, Rovaris K, Haiter F (2016). Impact of micronutrients supplementation on bone repair around implants: microCT and counter-torque analysis in rats. J Appl Oral Sci.

[34] Nakamura Y, Hayashi K, Abu-Ali S, Naito M, Fotovati A (2008). Effect of preoperative combined treatment with alendronate and calcitriol on fixation of hydroxyapatite-coated implants in ovariectomized rats. J Bone Jt Surg Am.

[35] Kwiatek J, Jaroń A, Trybek G. Impact of the 25-hydroxycholecalciferol concentration and vitamin D deficiency treatment on changes in the bone level at the implant site during the process of osseointegration: a prospective, randomized, controlled clinical trial. J Clin Med 2021;10(3). Verfügbar unter: https://www.ncbi.nlm.nih.gov/pmc/articles/PMC7867129/. 10.3390/jcm10030526 PMC7867129 33540512

[36] Guido Mangano F, Ghertasi Oskouei S, Paz A, Mangano N, Mangano C (2018). Low serum vitamin D and early dental implant failure: Is there a connection? A retrospective clinical study on 1740 implants placed in 885 patients. J Dent Res Dent Clin Dent Prospects.

[37] Flanagan D, Mancini M (2015). Bimaxillary full arch fixed dental implant supported treatment for a patient with renal failure and secondary hyperparathyroidism and osteodystrophy. J Oral Implantol.

[38] Bryce G, MacBeth N (2014). Vitamin D deficiency as a suspected causative factor in the failure of an immediately placed dental implant: a case report. J R Nav Med Serv.

[39] Fretwurst T, Grunert S, Woelber JP, Nelson K, Semper-Hogg W. Vitamin D deficiency in early implant failure: two case reports. Int J Implant Dent. 2020;2. Verfügbar unter: https://www.ncbi.nlm.nih.gov/pmc/articles/PMC5124022/. 10.1186/s40729-016-0056-0 PMC5124022 27888492

[40] Lips P, Hosking D, Lippuner K, Norquist JM, Wehren L, Maalouf G (2006). The prevalence of vitamin D inadequacy amongst women with osteoporosis: an international epidemiological investigation. J Intern Med.

[41] Chapuy MC, Schott AM, Garnero P, Hans D, Delmas PD, Meunier PJ (1996). Healthy elderly French women living at home have secondary hyperparathyroidism and high bone turnover in winter. EPIDOS Study Group. J Clin Endocrinol Metab.

[42] Greene-Finestone LS, Berger C, de Groh M, Hanley DA, Hidiroglou N, Sarafin K (2011). 25-Hydroxyvitamin D in Canadian adults: biological, environmental, and behavioral correlates. Osteoporos Int.

[43] Holick MF, Siris ES, Binkley N, Beard MK, Khan A, Katzer JT (2005). Prevalence of Vitamin D inadequacy among postmenopausal north american women receiving osteoporosis therapy. J Clin Endocrinol Metab.

[44] Holick MF (2006). High prevalence of vitamin D inadequacy and implications for health. Mayo Clin Proc.

[45] Charoenngam N, Shirvani A, Holick MF (2019). The ongoing D-lemma of vitamin D supplementation for nonskeletal health and bone health. Curr Opin Endocrinol Diab Obes..

[46] Fretwurst T, Wölber JP, Nelson K (2020). Vitamin D zur implantation—Ist ein Screening mit Substitution sinnvoll?. Quintessenz Zahnmedizin.

[47] Shahram G, Karl UV, Al-Maawi S (2020). Vitamin D als schlüsselelement für immunabwehr und regeneration. Implantol J..

[48] Irie MS, Rabelo GD, Spin-Neto R, Dechichi P, Borges JS, Soares PBF (2018). Use of micro-computed tomography for bone evaluation in dentistry. Brazil Dental J..

[49] Vandeweghe S, Coelho PG, Vanhove C, Wennerberg A, Jimbo R (2013). Utilizing micro-computed tomography to evaluate bone structure surrounding dental implants: a comparison with histomorphometry. J Biomed Mater Res B Appl Biomater.

[50] Max-Bergmann-Center for Biomaterials, Technische Universität Dresden, Budapester Str. 27, 01062 Dresden, Germany, Bernhardt R, Kuhlisch E, Schulz M, Eckelt U, Stadlinger B. Comparison of bone-implant contact and bone-implant volume between 2D-histological sections and 3D-SRµCT slices. ECM. 2012;23:237–48. 10.22203/ecm.v023a18 22492016

